# Bromocriptine, Selegiline and Amantadine in the Treatment of Depression—A Systematic Review

**DOI:** 10.3390/ph18111664

**Published:** 2025-11-03

**Authors:** Rafał Bieś, Marek Krzystanek, Michał Górski, Agnieszka Koźmin-Burzyńska, Anna Warchala, Beata Trędzbor, Ewa Martyniak, Joanna Fojcik

**Affiliations:** 1 Department and Clinic of Psychiatric Rehabilitation, Doctoral School, Faculty of Medical Sciences, Medical University of Silesia in Katowice, 40-635 Katowice, Poland; 2 Department and Clinic of Psychiatric Rehabilitation, Faculty of Medical Sciences, Medical University of Silesia in Katowice, 40-635 Katowice, Poland; 3 Department of Clinical and Community Psychiatry and Psychology, Medical Faculty, WSB University, Cieplaka 1C, 41-300 Dąbrowa Górnicza, Poland; 4 Department of Chronic Diseases and Civilization-Related Hazards, Faculty of Public Health in Bytom, Medical University of Silesia in Katowice, 40-055 Katowice, Poland; 5 Department of Psychiatry, Department of Neurology, Faculty of Health Sciences in Katowice, Medical University of Silesia in Katowice, 40-635 Katowice, Poland

**Keywords:** dopaminergic antidepressants, treatment-resistant depression, motivational deficits, anhedonia, bromocriptine, amantadine, selegiline, monoamine oxidase inhibitors, dopamine agonists, monoaminergic dysfunction, depression

## Abstract

**Background**: Depressive disorders are among the most common and disabling psychiatric conditions. A growing body of evidence suggests that dopaminergic dysfunction plays a key role in the pathophysiology of anhedonia, amotivation, and psychomotor slowing. This systematic review aims to determine whether bromocriptine, amantadine, and selegiline improve depressive symptoms compared to placebo or standard antidepressants, and to test the hypothesis that their antidepressant effects are mediated by dopaminergic modulation of motivational and reward circuits. **Methods**: The review followed PRISMA guidelines and was registered in PROSPERO. **Results**: Twenty-eight studies met inclusion criteria. Selegiline, particularly in transdermal form, reduced HAM-D scores by approximately 40% from baseline and by 25–30% relative to placebo. Amantadine augmentation achieved ≥50% symptom improvement in 60–70% of treatment-resistant cases. Bromocriptine yielded comparable response rates (~60%) to tricyclic antidepressants. All three agents improved anhedonia and motivational deficits. **Conclusions**: Findings suggest that bromocriptine, amantadine, and selegiline may represent effective dopaminergic antidepressants, particularly for treatment-resistant or atypical depression. Further large-scale, methodologically rigorous studies are needed to confirm their clinical utility.

## 1. Introduction

Depressive disorders cause substantial disability worldwide [1]. Standard pharmacotherapies mainly target serotonergic and noradrenergic systems and often do not relieve core motivational symptoms such as anhedonia and psychomotor slowing [2]. Dopaminergic dysfunction is increasingly implicated in these symptoms and in poor treatment response. Targeting dopamine may therefore help patients with predominant motivational deficits [3,4]. Several drugs with dopaminergic activity have been studied for depression. Examples include bupropion, pramipexole, and newer formulations such as the selegiline patch [5]. Systematic reviews and meta-analyses exist for some dopamine-targeting agents, but evidence is scattered across different drug classes.

The conventional antidepressant model, centered on serotonergic and noradrenergic deficits, does not fully explain symptoms such as anhedonia and motivational impairment. These symptom domains are increasingly attributed to dysfunction within the dopaminergic system. Altered activity in mesolimbic and mesocortical pathways has been repeatedly associated with impaired reward processing in depression [6]. Anhedonia, recognized as a core diagnostic criterion in DSM-5, encompasses not only a diminished capacity to experience pleasure but also a reduced willingness to exert effort and diminished consummatory reward response [7]. Neuroimaging studies have identified key structures involved in motivational and reward valuation processes, including the ventral tegmental area, nucleus accumbens, striatum, anterior cingulate cortex, and prefrontal cortex. Dysregulation within these circuits contributes to the emergence of anhedonic symptoms [8]. Notably, anhedonia shows limited responsiveness to treatment with selective serotonin reuptake inhibitors, and its baseline severity is a negative predictor of antidepressant response [9].

Bromocriptine is a potent D2 receptor agonist with high oral bioavailability and hepatic metabolism via CYP3A4. Its primary neuroanatomical targets include the mesolimbic and mesocortical pathways, where it enhances dopaminergic transmission, particularly in the ventral tegmental area (VTA) and nucleus accumbens [6]. Amantadine acts as an NMDA receptor antagonist and indirect dopamine agonist by promoting dopamine release and inhibiting its reuptake. It crosses the blood–brain barrier effectively, with renal elimination as the primary route. Selegiline is a selective monoamine oxidase B inhibitor that increases synaptic dopamine levels, particularly in the striatum and prefrontal cortex. In its transdermal form, it bypasses first-pass metabolism, reducing the risk of tyramine interaction. These agents exert their clinical effects mainly through modulation of dopaminergic signaling in the VTA–nucleus accumbens–prefrontal cortex circuit, which is central to reward processing, motivation, and mood regulation [8].

Because traditional antidepressants show limited efficacy against core depressive symptoms, interest in dopaminergic compounds has increased. The dopaminergic system plays a crucial role in reward processing, motivational drive, and goal-directed behavior. Pharmacological agents that modulate dopamine transmission, although initially developed for other medical indications, are increasingly considered potential therapeutic options for treatment-resistant depression [10]. Particular attention has been directed toward dopamine receptor agonists, selective monoamine oxidase B inhibitors, and modulators of dopamine and glutamate release. These compounds exhibit favorable properties in enhancing motivation and promoting goal-directed activity. Both preclinical data and preliminary clinical findings indicate that dopaminergic agents may alleviate anhedonia and motivational deficits, particularly in patients who show poor response to conventional antidepressant therapies. However, the current body of evidence remains limited, methodologically heterogeneous, and often constrained by insufficient statistical power [11].

Despite growing interest in dopaminergic agents as potential treatments for depression, there is currently no comprehensive systematic synthesis of evidence evaluating the efficacy and mechanisms of bromocriptine, amantadine, and selegiline in this context. This review aims to address this gap by summarizing and critically analyzing available clinical studies on these three agents. Given the potential adverse effects, such as impulsivity and psychotic symptoms resulting from excessive dopaminergic activation, further systematic investigation of this pharmacological group is warranted. This issue is particularly important in depressive syndromes characterized by predominant anhedonia. Such an approach is consistent with the concept of personalized psychiatry, which seeks to adapt treatment strategies to each patient’s individual clinical, neurobiological, and pharmacogenetic profile [12]. Identifying depressive phenotypes that reflect dysfunction within specific monoaminergic systems, and implementing targeted serotonergic, noradrenergic, or dopaminergic interventions, may improve therapeutic efficacy while reducing the risk of adverse effects. Despite individual reviews of single dopamine-targeting agents, there is no comprehensive systematic synthesis that focuses on bromocriptine, amantadine, and selegiline together. This review fills that gap. We include studies of both unipolar and bipolar depression. We note that evidence strength differs across agents and that mechanisms may not generalize between unipolar and bipolar disorders. For context, recent systematic reviews have summarized evidence for dopamine agonists and other dopamine-targeting strategies [13].

## 2. Materials and Methods

The systematic review was conducted in accordance with the PRISMA (Preferred Reporting Items for Systematic Reviews and Meta-Analyses) guidelines [14] with the complete PRISMA checklist provided in the Supplementary Materials (File S1). The review protocol was registered in the PROSPERO registry (ID: CRD420251107504). The protocol specified qualitative synthesis only; quantitative meta-analysis was not planned due to heterogeneity of study designs and outcome measures.

### 2.1. Inclusion and Exclusion Criteria

This review included clinical studies investigating the use of bromocriptine, amantadine, or selegiline in the treatment of depression. Studies enrolling patients of all ages, including children, adolescents, and adults, were eligible regardless of sex. Trials addressing unipolar depression, bipolar depression, and treatment-resistant depression were considered. Only original research articles reporting the use of at least one of the three specified agents at therapeutic doses were included. Eligible studies had to specify the compound used, dosage, duration of treatment, and route of administration. Randomized controlled trials, open-label studies, prospective and retrospective studies, as well as case reports, were included. Studies involving patients with comorbid conditions, including psychiatric comorbidities, were not excluded provided that depression was the primary indication for treatment. Exclusion criteria included preclinical studies, research based solely on animal or in vitro models, narrative reviews, systematic reviews, and meta-analyses. Studies focusing on other therapeutic indications without a substantial emphasis on depressive symptoms were excluded. Articles without full-text access or published in languages other than English were also excluded. Both unipolar and bipolar depressive episodes were eligible for inclusion; when possible, we report findings separately because pathophysiology and response to dopaminergic interventions may differ between these groups.

### 2.2. Search Strategy

The PubMed database was searched using the following Boolean query: (“depressive disorder” [MeSH Terms] OR “depressive episode” OR “major depression” OR “bipolar depression” OR “recurrent depression”) AND (“bromocriptine” OR “amantadine” OR “selegiline”). Equivalent search strategies were adapted for Scopus, Embase, Web of Science, Cochrane Library, and the ClinicalTrials.gov registry. The time range was not restricted, as our aim was to include all available clinical studies regardless of publication date. This approach ensured the most comprehensive coverage possible. Since we did not apply date filters, specifying a time limit was not relevant to our search objectives. The search was limited to full-text articles published in English. Additional databases searched included Scopus, Embase, Cochrane Library, Web of Science, and the ClinicalTrials.gov registry. The first 200 results from Google Scholar were also screened based on title and abstract. No additional studies meeting the inclusion criteria were identified outside of the major databases.

### 2.3. Eligible Studies

**Figure 1:**
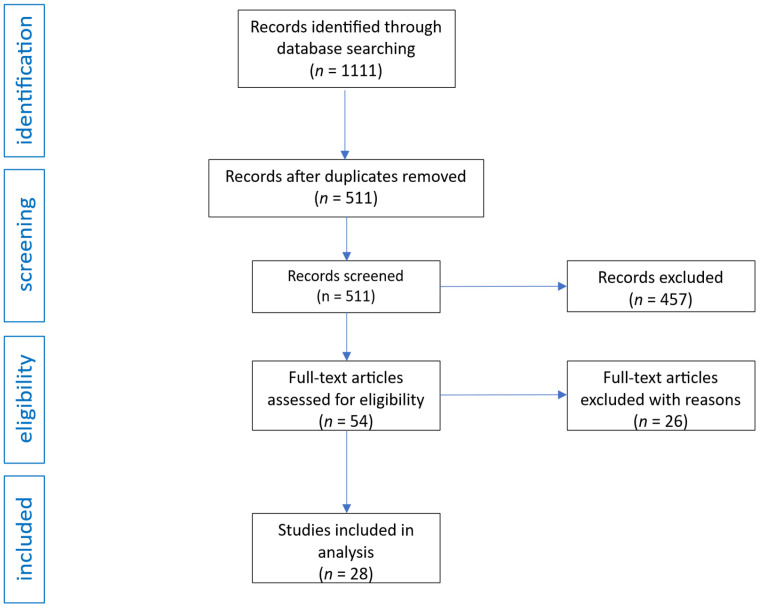
Flow diagram of study analysis and selection for review.

The initial search yielded 1111 records. After the removal of 600 duplicates, 511 unique publications remained. These were screened by title and abstract, resulting in the exclusion of 457 records that did not meet inclusion criteria. Fifty-four articles were selected for full-text review. Of these, 26 were excluded due to lack of relevant outcomes or insufficient relevance to the review topic. Ultimately, 28 studies met all eligibility criteria and were included in the final analysis. The selection process is presented in Figure 1.

### 2.4. Data Extraction

From each included study, key information was extracted, including the first author’s name, study design, dopaminergic agent used, sample size and participant characteristics, intervention details, and methodological quality assessment. Particular attention was paid to the characteristics of the study population, the type of depression, and the method of outcome measurement. The extracted data were compiled in a manner that allows for cross-study comparison and are presented in Table 1, Table 2 and Table 3.

### 2.5. Data Quality

Methodological quality was assessed using the Quality Assessment Tool for Quantitative Studies (QATQS), developed by the Effective Public Health Practice Project [43,44]. This tool evaluates eight key domains: study design, risk of selection bias, confounders, blinding, data collection methods, withdrawals and dropouts, intervention integrity, and data analysis. Each domain is rated on a three-point scale: 1—strong; 2—moderate; 3—weak. A study was classified as strong if no domain received a weak rating, moderate if one domain was rated as weak, and weak if two or more domains received a weak rating. Two reviewers independently evaluated the quality of each study. Of the 28 studies included, 9 were rated as strong, 12 as moderate, and 7 as weak according to the QATQS criteria. Weak ratings most frequently involved blinding and confounder control, primarily due to the predominance of open-label or small-sample studies. Conversely, data collection methods and study design were most frequently rated as strong. The detailed quality ratings for each study are summarized in Table 1, Table 2 and Table 3. Potential confounders, including psychiatric and somatic comorbidities, were considered during data synthesis by qualitatively describing their presence and evaluating their potential influence on treatment outcomes in each study. Studies were not excluded based on comorbidities but these variables were discussed when relevant to the interpretation of results. Their assessments were consistent, and no further adjudication was required. Quality ratings and detailed extracted data are presented in Table 1, Table 2 and Table 3.

## 3. Results

### 3.1. The Effect of Bromocriptine on Depression

Bromocriptine has demonstrated potential antidepressant properties, as shown in several clinical studies. In a comparative trial with imipramine involving patients with endogenous depression, bromocriptine administered at a mean dose of 34 mg per day reduced Hamilton Depression Rating Scale (HDRS) scores from 19.9 to 7.8 after six weeks of treatment [15]. In another double-blind study comparing bromocriptine with amitriptyline in 83 patients, both agents produced comparable reductions in depressive symptoms, with no statistically significant differences between treatment groups. Bromocriptine may be particularly beneficial for patients requiring alternative therapies with minimal sedative or anticholinergic effects [16]. In an open-label study of patients with depression, bromocriptine administered at doses ranging from 20 to 60 mg per day resulted in near-complete remission in 9 of 12 participants within five weeks. The observed reduction in cerebrospinal fluid (CSF) homovanillic acid (HVA) levels confirmed the drug’s dopaminergic activity; however, symptom improvement correlated more strongly with noradrenergic markers, suggesting a possible involvement of the noradrenergic system in its antidepressant effect [17].

Bromocriptine has also been investigated as an augmentation strategy in treatment-resistant depression (TRD). In a six-week pilot study involving patients unresponsive to tricyclic or heterocyclic antidepressants, four of six participants achieved at least a 50% improvement in HDRS scores, with noticeable benefits emerging after approximately four weeks of treatment. The most pronounced improvements were observed in sleep disturbances, mood, anxiety, and psychomotor retardation [10]. Additional evidence supporting bromocriptine’s efficacy comes from case reports describing four patients with organic abulia and depressive symptoms who experienced significant clinical improvement with bromocriptine administered at doses ranging from 25 to 70 mg per day [18].

A clinical trial registered on ClinicalTrials.gov (NCT00704860) investigated the effects of treatment-resistant depression therapy on hippocampal volume and serotonin gene expression, with bromocriptine included as one of the experimental agents. The study enrolled 27 patients with TRD who received open-label treatment consisting of individually selected antidepressants (tricyclics, selective serotonin reuptake inhibitors, serotonin-norepinephrine reuptake inhibitors, monoamine oxidase inhibitors, lithium, and atypical antipsychotics), while bromocriptine and pramipexole were used as augmentation options in selected cases. The primary endpoints included changes in hippocampal volume assessed by magnetic resonance imaging (MRI) at baseline and after either six months of sustained remission or twelve months in nonresponders, as well as analyses of serotonin-related gene polymorphisms (5-HT1A G-1019, 5-HTTLPR, and TPH*A) and their associations with clinical outcomes. The study was based on the hypothesis that patients with TRD exhibit reduced hippocampal volume compared with healthy controls, and that treatment—particularly bromocriptine augmentation—may partially reverse hippocampal atrophy by enhancing dopaminergic transmission and promoting neuroplasticity. It was also proposed that the G-1019 genotype of the 5-HT1A receptor might be associated with poorer treatment response and greater hippocampal atrophy, whereas the long allele of 5-HTTLPR might predict more favorable clinical outcomes. In this trial, bromocriptine was administered to selected patients to enhance prefrontal dopaminergic transmission, improve depressive and cognitive symptoms, and potentially mitigate hippocampal atrophy, a frequent finding in chronic TRD. Although the study results were not published in the registry, limiting interpretation of its clinical outcomes, the trial reflects a growing interest in bromocriptine as an augmentation strategy in TRD, particularly in the context of chronic depression associated with hippocampal structural changes.

Regarding safety, bromocriptine was generally well tolerated. The most common adverse effects included nausea, dizziness, and headache, consistent with the known dopaminergic agonist profile [15]. No serious adverse events or manic switches were reported among patients with unipolar depression across the reviewed studies. Nevertheless, caution remains warranted due to the potential risk of mania induction in individuals with bipolar disorder [10].

### 3.2. The Effect of Amantadine on Depression

Amantadine (AMA), primarily recognized as an *N*-methyl-D-aspartate (NMDA) receptor antagonist and modulator of the dopaminergic system, has shown promise as an adjunctive agent in the treatment of depression, particularly in treatment-resistant cases. Its mechanism of action involves enhancement of presynaptic dopamine release, inhibition of dopamine reuptake, and modulation of glutamatergic transmission, which together may contribute to the improvement of depressive symptoms and psychomotor activation [28]. Clinical data indicate that amantadine at a dose of 200 mg per day can prevent the exacerbation of depressive symptoms induced by interferon therapy in patients with hepatitis C virus (HCV) infection [27]. Moreover, amantadine has been shown to mitigate the decline in quality of life among patients treated with interferon and ribavirin [26]. In individuals with cocaine use disorder, amantadine significantly reduced depressive symptoms, improved treatment adherence, and diminished drug craving [25].

Particularly relevant findings have emerged from studies on treatment-resistant depression (TRD) [20]. In a randomized controlled trial involving patients with well-defined unipolar TRD who had not responded to imipramine (100–150 mg per day), adjunctive administration of amantadine (100–150 mg per day) for six weeks produced a significant reduction in depressive symptoms, as measured by the Hamilton Depression Rating Scale (HDRS) and Beck Depression Inventory (BDI). Imipramine monotherapy did not result in comparable improvement. Notably, the therapeutic benefits of amantadine persisted after discontinuation, and pharmacokinetic analyses revealed no significant interaction between amantadine and imipramine [19]. These findings were supported by a subsequent study involving a larger sample (*n* = 50), in which augmentation of imipramine (100 mg per day) with amantadine led to a significantly greater reduction in HDRS scores after six weeks compared with imipramine alone [21]. Amantadine has also demonstrated efficacy in patients with catatonic symptoms associated with bipolar depression, where treatment produced rapid and sustained remission of both depressive and catatonic features [22].

Growing interest in amantadine as an augmentation strategy for TRD has led to the initiation of a randomized clinical trial (NCT04936126) comparing amantadine (200 mg per day) with pramipexole and quetiapine as adjuncts to sertraline in 150 patients with TRD. The study aims to evaluate not only the efficacy of these agents in reducing depressive symptoms but also their impact on levels of neurotrophic factors, including brain-derived neurotrophic factor (BDNF) and nerve growth factor (NGF), after eight weeks of treatment. Importantly, amantadine has also shown beneficial effects in bipolar depression. In one report, four patients with bipolar I disorder, resistant to at least two antidepressants, received amantadine (100–200 mg per day), which produced rapid improvement in depressive symptoms within one week, without adverse effects or induction of mania. Treatment was discontinued two weeks after symptom remission, and no relapse occurred [28]. Similar results were observed in a 67-year-old woman with bipolar I disorder and Borna disease virus (BDV) infection, in whom addition of amantadine (titrated up to 200 mg per day) to ongoing treatment led to marked improvement between days 8 and 11, with full remission achieved by day 15 [24]. These outcomes were further supported by an open-label prospective study involving 30 patients with depression and BDV infection, in which 19 individuals demonstrated a clinical antidepressant response correlated with reduced infection severity [23].

Amantadine, administered at doses ranging from 100 to 300 mg per day, may represent an effective and relatively safe augmentation strategy for patients with TRD, depression associated with somatic comorbidities (such as HCV infection), and catatonia. Available data suggest a rapid onset of action, absence of significant pharmacokinetic interactions, and a favorable tolerability profile. However, further double-blind and adequately powered clinical studies are required to confirm these preliminary findings.

### 3.3. The Effect of Selegiline on Depression

Selegiline is a selective monoamine oxidase B (MAO-B) inhibitor that, at higher doses, also affects monoamine oxidase A (MAO-A) activity in the central nervous system, thereby exhibiting potential antidepressant properties. Its transdermal formulation, known as the selegiline transdermal system (STS), offers distinct advantages by bypassing the gastrointestinal tract and reducing the risk of tyramine-related interactions [29,30,35]. In short-term clinical studies involving adults with major depressive disorder (MDD), STS produced significant reductions in depressive symptoms as measured by the Hamilton Depression Rating Scale (HAMD) and the Montgomery–Åsberg Depression Rating Scale (MADRS) [31,32], with noticeable improvement observed as early as the first week of treatment. Long-term studies further demonstrated that STS reduces the risk of depressive relapse and prolongs the time to recurrence [32,33].

STS has shown efficacy across several subtypes of depression. In atypical depression, it alleviates hypersomnia, hyperphagia, and interpersonal anxiety, likely due to its dopaminergic and serotonergic effects [29,37]. In treatment-resistant depression (TRD), including cases in which patients decline conventional pharmacotherapy, STS—administered either as monotherapy or in combination with agents such as ketamine—has produced rapid and sustained clinical improvement [34,40]. STS may therefore represent a viable treatment option for patients who do not tolerate selective serotonin reuptake inhibitors (SSRIs), tricyclic antidepressants (TCAs), or classical monoamine oxidase inhibitors (MAOIs) [32,37]. However, in adolescent populations aged 12 to 17 years, STS has not demonstrated superiority over placebo, despite good tolerability [36], indicating that its efficacy in this age group remains uncertain. Selegiline has also been explored in bipolar depression, where it may exert mood-stabilizing effects, although further evidence is required [42]. When comparing routes of administration, the transdermal formulation of selegiline is generally safer than oral MAOIs and TCAs. It is associated with fewer systemic adverse effects, a lower incidence of sexual dysfunction, and reduced cardiovascular complications. The most common side effects are local skin reactions at the application site, which are typically mild [39]. At a dose of 6 mg per 24 h, STS does not require dietary tyramine restrictions, thereby enhancing treatment adherence and patient comfort [31,33].

Selegiline may also be used in combination therapy with 5-hydroxytryptophan (5-HTP), particularly in patients who do not tolerate standard antidepressant regimens or who exhibit only partial response. This strategy may be especially beneficial in individuals with prominent anergia and psychomotor retardation, where the combined enhancement of dopamine and serotonin availability contributes to increased energy and motivation [38]. In both unipolar and bipolar depression, the combination of selegiline and 5-HTP has been shown to produce significantly greater antidepressant effects than 5-HTP alone. In atypical depression, selegiline’s mechanism of action may explain its efficacy in patients unresponsive to other antidepressants, particularly those presenting with prominent anxiety, somatic symptoms, and early-onset illness [29]. Clinical observations suggest that doses exceeding 10 mg per day result in superior outcomes compared with placebo, while maintaining an acceptable safety profile [41,42].

Data from ClinicalTrials.gov further support the efficacy and safety of transdermal selegiline in depression. In trial NCT00531947, STS administered at 6 mg per 24 h significantly reduced the risk of relapse of major depressive episodes compared with placebo (16.8% versus 30.7%) over 52 weeks of maintenance therapy, with a favorable tolerability profile and no need for dietary restrictions. In another short-term randomized trial using flexible dosing (6–12 mg per 24 h), STS demonstrated greater efficacy than placebo in reducing depressive symptoms according to HAMD-28 and MADRS scores, although some therapeutic effects were limited by adverse events (NCT00023274). Conversely, in a study of adolescents with moderate to severe depression (NCT00532773), STS did not show a significant advantage over placebo, underscoring the need for caution in this population and highlighting the necessity for further research.

Overall, current evidence supports the efficacy of STS in adult patients with major depressive disorder and its potential role in the long-term management of depression, particularly among individuals at high risk of relapse or those who are intolerant to conventional antidepressant therapies.

## 4. Discussion

This systematic review evaluated studies retrieved from medical databases concerning the potential efficacy of dopaminergic agents in the treatment of depression. Selegiline has demonstrated effectiveness in reducing depressive symptoms, as measured by the Hamilton Depression Rating Scale (HAMD) and Montgomery–Åsberg Depression Rating Scale (MADRS), in adults with major depressive disorder. It has also shown efficacy in preventing relapse, particularly in patients with atypical or chronic forms of depression [29,30,31,32,33]. Amantadine, when used as an augmentation strategy alongside antidepressant therapy, has been associated with a significant reduction in depressive symptoms among patients with treatment-resistant depression [19,21,26,27]. Bromocriptine has shown comparable efficacy to tricyclic antidepressants in the treatment of endogenous depression and exhibits potential as an augmentation therapy in treatment-resistant cases [10,15,16,17]. All three agents exert beneficial effects on core depressive symptoms such as anhedonia, anergia, psychomotor retardation, and low mood. These findings support the hypothesis that dopaminergic dysfunction plays a key role in the pathophysiology of depression, particularly in its chronic and treatment-resistant forms.

The antidepressant properties of dopaminergic agents likely derive from the interplay between dopamine and glutamate neurotransmission within the mesolimbic reward circuit, particularly the VTA–nucleus accumbens–prefrontal cortex pathway. As described in the Introduction, bromocriptine enhances D2-mediated signaling, while amantadine modulates both dopamine and NMDA receptor activity, and selegiline increases synaptic dopamine via MAO-B inhibition. This dopaminergic–glutamatergic crosstalk may underlie improvements in motivation and anhedonia. Alterations in these circuits are central to the neurobiology of depression, and their modulation represents a key mechanistic target for dopaminergic antidepressants.

The findings confirm the increasing importance of the dopaminergic system in the treatment of depression, particularly in cases resistant to standard therapy and characterized by predominant symptoms of anergia, psychomotor retardation, and anhedonia. Consistent with previous evidence, deficits in dopaminergic transmission within the prefrontal cortex and mesolimbic pathways may contribute to the persistence of depressive symptoms. Pharmacological agents that enhance dopaminergic activity may help reverse these neurobiological alterations and alleviate clinical manifestations of depression [45,46]. Although this review focuses on bromocriptine, amantadine, and selegiline, it is important to acknowledge other dopaminergic agents with established or potential antidepressant properties. Among them, bupropion (a norepinephrine-dopamine reuptake inhibitor) has long been approved and widely used as an antidepressant [11,13]. In addition, several medications indicated for attention-deficit/hyperactivity disorder (ADHD), such as methylphenidate and lisdexamfetamine, exert dopaminergic effects that can influence mood and motivation. However, because these agents have distinct pharmacological profiles, regulatory indications, and a substantial body of prior literature, they were not included in the present analysis, which is specifically limited to dopaminergic drugs primarily developed for other medical indications and subsequently evaluated for antidepressant effects.

It is important to note that the strength of the available evidence varies considerably among the compounds analyzed. The antidepressant efficacy of selegiline is supported primarily by multiple randomized controlled trials, providing higher-quality evidence and greater generalizability. In contrast, data on amantadine and bromocriptine are derived mainly from open-label studies and individual case reports, which, while valuable in generating hypotheses, offer limited inferential strength. Consequently, findings regarding these two agents should be interpreted as preliminary and warrant confirmation in large, placebo-controlled studies. This distinction underscores that the present review integrates heterogeneous forms of evidence and that the overall conclusions should be viewed as exploratory rather than definitive. A formal meta-analytic approach was not applied because of substantial heterogeneity in study design, outcome measures, and reporting standards among the included trials. However, we recognize that quantitative synthesis may be feasible in the future as more standardized data become available, particularly for selegiline. This distinction highlights that the conclusions drawn from randomized controlled trials (e.g., selegiline) carry greater evidential weight than those from open-label studies or individual case reports, which remain hypothesis-generating.

Selegiline, especially in transdermal form, shows efficacy in atypical depression, often linked to fatigue, hypersomnia, and increased appetite [47]. The transdermal form minimizes the risk of tyramine interactions, which enhances safety and treatment comfort [30,31,33]. Amantadine, as a modulator of dopaminergic and glutamatergic systems, may act rapidly and effectively in patients with TRD, as confirmed by studies assessing its role in augmenting classical antidepressants [19,21,48]. The antidepressant potential of amantadine in TRD and in patients with comorbidities such as hepatitis C or cocaine dependence is particularly noteworthy. Mechanistically, amantadine acts both as a weak NMDA receptor antagonist and an indirect dopamine agonist, enhancing dopamine release while inhibiting its reuptake. This dual dopaminergic–glutamatergic modulation may underlie its ability to improve motivation and psychomotor activity, differentiating it from classical antidepressants. Further mechanistic studies are warranted to clarify the relative contributions of these two neurotransmitter systems to its antidepressant action. Although the number of studies is limited, bromocriptine has demonstrated efficacy comparable to that of tricyclic antidepressants. Owing to its low sedative and anticholinergic profile, it may represent a valuable treatment option for patients who poorly tolerate other classes of antidepressants [15,16]. These findings further support the hypothesis that augmentation with dopaminergic agents may help reverse certain neurobiological alterations observed in depression, including reduced prefrontal cortex activity and impairments in reward processing [49]. Although bromocriptine demonstrated antidepressant effects comparable to tricyclic antidepressants, these findings originate from small-sample studies conducted several decades ago, often with methodological constraints by today’s standards. Consequently, while these results are encouraging, their direct translation to current clinical practice remains limited.

The use of dopaminergic drugs, especially in bipolar depression, carries a potential risk of mania induction. This also applies to patients with undiagnosed bipolar affective disorder, diagnosed as having a depressive episode or recurrent depression. In the analyzed studies, no cases of manic episodes or manic switch were reported, but these data are mainly based on small clinical samples [10,15,40]. Therefore, caution and appropriate patient selection are necessary before introducing dopaminergic drugs in treatment-resistant depression. Although no manic switches were reported in the reviewed studies, the available data are limited by small sample sizes and heterogeneous populations. Therefore, the absence of observed manic episodes should not be interpreted as evidence of safety. Given the dopaminergic mechanism of action and off-label status of these treatments, careful patient selection and close clinical monitoring are warranted, especially in individuals with a personal or family history of bipolar disorder.

Regarding safety and tolerability, bromocriptine, amantadine, and selegiline are generally associated with a benign adverse event profile compared to classical MAOIs and TCAs, but specific risks must be considered. The most commonly reported adverse effects included nausea, headache, dizziness, and local skin reactions (for transdermal selegiline). Unlike TCAs, these drugs are typically associated with lower anticholinergic burden and less cardiovascular risk. However, dopaminergic activation carries a theoretical risk of impulsivity, agitation, and manic switch, which may be underdetected in small or open-label studies. Comparative safety data with SSRIs and other antidepressants remain limited. Table 4 summarizes the main adverse events and tolerability considerations reported across the included studies.

Increasing the number of dopaminergic drugs available for treating depression with dopaminergic symptom clusters may enhance therapeutic options for patients. This is particularly feasible in personalized pharmacotherapy of depression, where drug mechanism selection is tailored to the symptom cluster [13]. However, the introduction and registration of new dopaminergic drugs for depression treatment require clinical trials with active comparators demonstrating their efficacy, including in treatment-resistant depression. It is important to emphasize that dopaminergic strategies in depression extend beyond the three compounds analyzed in this review. D2/D3 receptor partial agonists such as cariprazine, brexpiprazole, and aripiprazole have shown antidepressant and pro-motivational effects in clinical studies, often as augmentation agents. Their development highlights the growing interest in targeting the dopaminergic system in a more selective and receptor-specific manner. Recent randomized controlled trials and meta-analyses have confirmed the antidepressant efficacy of these agents as augmentation strategies in major depressive disorder [50,51,52]. These studies provide comparative evidence supporting the receptor-specific dopaminergic modulation achieved by cariprazine, brexpiprazole, and aripiprazole. In this context, bromocriptine, amantadine, and selegiline remain clinically relevant, particularly for patients with treatment-resistant or atypical depression, and may complement newer dopaminergic treatments. Among the three agents analyzed, selegiline (particularly in its transdermal formulation) has the strongest empirical support, based on multiple large-scale randomized controlled trials. In contrast, evidence for bromocriptine and amantadine remains limited to smaller open-label or early-generation trials. Therefore, current clinical recommendations should favor selegiline in cases of dopaminergic or atypical depression, while bromocriptine and amantadine should be regarded as experimental or adjunctive options pending further controlled studies.

This systematic review has several important limitations that should be considered when interpreting the results. First, the included studies are characterized by heterogeneity in patient populations regarding age, illness duration, symptom severity, and previous treatment regimens, which may affect the generalizability of findings. For selegiline, most data come from studies involving adults with MDD, while results concerning adolescent populations remain inconclusive [36]. Additionally, some studies on amantadine and bromocriptine employed open-label designs or had small sample sizes, which limits statistical power and increases the risk of bias [10,15,17,19,21]. Additionally, potential publication bias should be considered, as positive findings are more likely to be published than negative or null results, which may overestimate the perceived efficacy of these agents. Furthermore, the scarcity of head-to-head comparative studies limits the ability to draw firm conclusions regarding the relative effectiveness of bromocriptine, amantadine, and selegiline compared to standard antidepressants or to one another. For selegiline, 52-week data are available, but they mainly concern maintenance therapy in MDD patients, with no possibility to extrapolate results to other patient groups such as those with bipolar depression or chronic treatment-resistant depression [32,33].

Dopaminergic drugs, including selegiline, amantadine, and bromocriptine, may constitute valuable therapeutic options in depression treatment. Despite limitations, the results of this analysis highlight the need for further well-designed clinical trials evaluating the efficacy and safety of these drugs. Further well-designed clinical trials are necessary to definitively assess the efficacy and safety of dopaminergic drugs in depression treatment. Future studies should focus on identifying specific depressive phenotypes and neurobiological markers associated with dopaminergic dysfunction, which could guide personalized pharmacotherapy. Comparative trials evaluating dopaminergic agents against standard antidepressants and other augmentation strategies are also warranted. Moreover, exploring the molecular mechanisms underlying their antidepressant effects, such as modulation of BDNF, neuroplasticity, and inflammatory pathways may provide important insights into novel therapeutic targets for treatment-resistant depression.

## 5. Conclusions

This systematic review shows that dopaminergic agents such as bromocriptine, amantadine and selegiline may be valuable additions to depression treatment. These drugs may be especially helpful in patients with anergia, amotivation and anhedonia. Among them, transdermal selegiline has the strongest evidence. Bromocriptine and amantadine show promising but preliminary results from open-label studies and case reports. From a clinical point of view, dopaminergic strategies could be most relevant for subtypes of depression with motivational and reward-processing deficits. Future studies should define such dopaminergic depression subtypes using multimodal methods. These should include clinical assessment, dopaminergic neuroimaging and functional tests of reward circuits. Further work should focus on biomarker-guided and pharmacogenetic predictors of response. Variants affecting dopamine receptor function or dopamine transporter activity may help personalize therapy. Preclinical and translational studies should also explore how dopaminergic drugs influence neurotrophic factors such as BDNF and NGF. Understanding these mechanisms could support the development of personalized treatments for dopaminergic dysfunction in treatment-resistant depression.

## Figures and Tables

**Table 1:** Summary of studies on the use of bromocriptine in the treatment of depression. This table includes the names of authors, study types, the dopaminergic drug used, participant characteristics, the intervention applied (dosage, treatment duration, comparative therapies), and the methodological quality assessment (QATQS).

Author	Study Type	Dopaminergic Drug	Participants	Intervention	QATQS
Waehrens et al. [15]	Double-blind randomized controlled trial	Bromocriptine	33 outpatients with unipolar depression	6-week treatment: bromocriptine (10–60 mg/day) vs. imipramine (75–250 mg/day)	1
Teohar et al. [16]	Double-blind multicenter randomized controlled trial	Bromocriptine	83 patients with unipolar depression	Bromocriptine (30–50 mg) vs. amitriptyline (120–200 mg)	1
Nordin et al. [17]	Open clinical trial	Bromocriptine	15 patients with depression; 12 completed 5-week treatment	Bromocriptine 20–60 mg/day for 5 weeks	3
Inoue et al. [10]	Open-label pilot study	Bromocriptine	6 patients with treatment-resistant depression	Bromocriptine (15–52.5 mg/day)	3
Barrett et al. [18]	Case series	Bromocriptine	4 patients with organic abulia and depressive symptoms	Bromocriptine (25–70 mg/day)	3

**Table 2:** Summary of studies on the use of amantadine in the treatment of depression. This table includes the names of authors, study types, the dopaminergic drug used, participant characteristics, the intervention applied (dosage, treatment duration, comparative therapies), and the methodological quality assessment (QATQS).

Author	Study Type	Dopaminergic Drug	Participants	Intervention	QATQS
Rogóz et al. [19]	Open-label prospective study	Amantadine	12 patients with treatment-resistant unipolar depression	Imipramine 100–150 mg/day for 6 weeks, then imipramine + amantadine 100–150 mg/day for 6 weeks	2
Stryjer et al. [20]	Open-label prospective study	Amantadine	8 patients with treatment-resistant depression	Amantadine up to 300 mg/day for 4 weeks as augmentation to ongoing antidepressants	2
Rogóz et al. [21]	Prospective controlled study (non-randomized)	Amantadine	50 patients with treatment-resistant unipolar depression	Group 1: imipramine 100 mg/day for 12 weeks; Group 2: imipramine 100 mg/day for 6 weeks, then imipramine + amantadine 150 mg/day for 6 weeks	2
Ene-Stroescu et al. [22]	Case report	Amantadine	58-year-old female with bipolar disorder and catatonia	Addition of amantadine to carbamazepine and lorazepam; rapid improvement in catatonia and depressive symptoms	3
Bode et al. [23]	Open-label prospective study	Amantadine	30 patients with depression and BDV infection	Oral amantadine for 8–12 weeks; antidepressant response in 19/30 patients; correlated with BDV reduction	2
Bode et al. [24]	Case report	Amantadine	67-year-old woman with BD-I, resistant depression, BDV infection	Amantadine (50–100 mg/day for 3 days, 200 mg/day for 6 weeks, 100 mg/day for 14 weeks, then 200 mg/day); dramatic symptom improvement from days 8–11, remission from day 15	3
Ziedonis et al. [25]	Randomized placebo-controlled trial (RCT)	Amantadine	Cocaine-dependent patients with depression receiving methadone	12-week treatment: placebo vs. amantadine vs. desipramine	1
Kronenberger et al. [26]	Randomized double-blind placebo-controlled trial	Amantadine	267 patients with chronic hepatitis C treated with IFN-alpha + ribavirin	Amantadine (200 mg/day) vs. placebo for 48 weeks; quality of life assessed	1
Quarantini et al. [27]	Randomized controlled pilot study	Amantadine	14 patients with hepatitis C treated with pegIFN + ribavirin	Amantadine (200 mg/day) vs. no amantadine for 24 weeks	2
Krzystanek et al. [28]	Case series	Amantadine	4 patients with BD-I resistant to ≥2 antidepressants	1 woman: 100 mg/day; 3 men: 200 mg/day	3

**Table 3:** Summary of studies on the use of selegiline in the treatment of depression. This table includes the names of authors, study types, the dopaminergic drug used, participant characteristics, the intervention applied (dosage, treatment duration, comparative therapies), and the methodological quality assessment (QATQS).

Author	Study Type	Dopaminergic Drug	Participants	Intervention	QATQS
Pae, Chi-Un et al. [29]	Randomized controlled trial	Transdermal selegiline	1289 patients (352 with atypical depression)	STS (3–12 mg/24 h) vs. placebo for several weeks	1
Bodkin et al. [30]	Randomized, double-blind, placebo-controlled, 6-week parallel study	Transdermal selegiline	177 adults (18–65 years), MDD (DSM-IV), 60% women, 93% White, 68% recurrent	20 mg/day STS patch (20 cm^2^), daily for 6 weeks	1
Amsterdam et al. [31]	Randomized, double-blind, placebo-controlled, 8-week study	Transdermal selegiline	289 adults (18–65 years), MDD (DSM-IV), HAMD-17 ≥ 20	20 mg/day STS (20 cm^2^), daily for 8 weeks	2
Jang et al. [32]	Randomized, double-blind, placebo-controlled 52-week study (maintenance phase)	Transdermal selegiline	322 patients in remission after 10 weeks STS 6 mg/24 h, MDD (DSM-IV)	STS 6 mg/24 h vs. placebo for 52 weeks	1
Amsterdam et al. [33]	Randomized, double-blind, placebo-substitution controlled, 52-week study	Transdermal selegiline	322 patients in remission after 10 weeks STS 6 mg/24 h, MDD (DSM-IV)	STS 6 mg/24 h vs. placebo for 52 weeks	1
Lu et al. [34]	Case series	Transdermal selegiline	6 patients (36–84 years), MDD, resistant to ≥3 antidepressants, ECT ineffective or declined	STS 6 mg/24 h + 1–3 IV ketamine infusions (0.5 mg/kg), for several days to 2 weeks	3
Feiger et al. [35]	Randomized, double-blind, controlled trial	Transdermal selegiline	265 patients with MDD (DSM-IV), no dietary restrictions	STS 6–12 mg/24 h for 8 weeks vs. placebo; outcomes: HAM-D28, MADRS, IDS-SR, Bech-6	2
DelBello et al. [36]	Randomized, double-blind, placebo-controlled trial	Transdermal selegiline	304 adolescents (12–17 years) with MDD (DSM-IV-TR), CDRS-R ≥ 45	STS 6–12 mg/24 h vs. placebo; assessments: CDRS-R, CGI-S, CGI-I	3
Quitkin et al. [37]	Open comparative study	Oral selegiline	17 patients with atypical depression	Selegiline for 6 weeks; 59% responders; doses often >10–20 mg/day; fewer side effects than classic MAOIs	3
Mendlewicz et al. [38]	Open-label phase + double-blind randomized phase	Oral selegiline	14 in open phase, 58 in RCT (18 deprenyl + 5-HTP, 21 5-HTP, 19 placebo); unipolar and bipolar depression	Selegiline + L-5-HTP vs. 5-HTP vs. placebo; greatest improvement in combined group; correlated with platelet MAO-B	3
Kim et al. [39]	Retrospective clinical analysis	Transdermal selegiline	117 adults, TRD (33 STS, 53 MAOI, 31 TCA)	STS 6 mg/24 h vs. oral MAOIs vs. TCA (1983–2015 data)	3
Higuchi et al. [40]	Case report	Oral selegiline	1 patient with TRD	Selegiline 7.5 mg/day orally	3
Mann et al. [41]	Randomized controlled trial	Oral selegiline	66 outpatients with depression	Selegiline: 10 mg/day (3 weeks), then 30 mg/day (3 weeks) vs. placebo	2
Mendlewicz et al. [42]	Randomized controlled trial	Oral selegiline	27 patients with depression (MDD: *n* = 22, BD: *n* = 5), HDRS ≥ 25	Selegiline 15 mg/day orally (5 mg 3×/day) vs. placebo	1

**Table 4:** This table includes the most commonly reported adverse events, serious adverse events, tolerability characteristics, and key safety considerations of bromocriptine, amantadine, and selegiline based on the studies included in the review. Comparative information regarding SSRIs, TCAs, and classical MAOIs is also provided to contextualize the safety profiles of these dopaminergic agents.

Drug	Common Adverse Events	Serious Adverse Events	Comparison to SSRIs/TCAs/MAOIs	Notable Safety Considerations
Bromocriptine	Nausea, dizziness, headache	Rare, none reported in reviewed studies	Less sedating and anticholinergic than TCAs	Risk of dopaminergic activation (mania, impulse control disorders)
Amantadine	Nausea, insomnia, agitation, mild GI symptoms	Rare psychiatric activation	Better tolerated than TCAs; limited data vs. SSRIs	Dopaminergic/glutamatergic activation may increase risk of agitation
Selegiline	Skin reactions at patch site, insomnia	Hypertensive crisis not reported at 6 mg/24 h	Fewer tyramine interactions than oral MAOIs	Mania risk in vulnerable individuals; higher doses require caution

Abbreviations: SSRI—selective serotonin reuptake inhibitors; TCA—tricyclic antidepressants; MAOI—monoamine oxidase inhibitors; GI—gastrointestinal.

## Data Availability

No new data were created or analyzed in this study. Data sharing is not applicable to this article.

## Supplementary Materials

The following supporting information can be downloaded at: https://www.mdpi.com/article/10.3390/ph18111664/s1, File S1: PRISMA checklist. Source: from [53].
