# Supplementary material for: Bromocriptine, Selegiline and Amantadine in the Treatment of Depression—A Systematic Review
Source: Pharmaceuticals (Basel). 2025 Nov 3;18(11):1664. doi: 10.3390/ph18111664 (PMC12655414; doi:10.3390/ph18111664)
Supplement: Supplementary file 1 [file pharmaceuticals-18-01664-s001.zip › PRISMA checklist.pdf]

PRISMA 2020 Checklist

Section and Topic	Item #	Checklist item	Location where item is reported
TITLE			
Title	1	Identify the report as a systematic review.	Title page – “Dopaminergic Drugs: Bromocriptine, Selegiline and Amantadine – in the Treatment of Depression. Systematic Review.”
ABSTRACT			
Abstract	2	See the PRISMA 2020 for Abstracts checklist.	Section titled “Abstract”, includes Background/Objectives, Methods, Results, and Conclusions.
INTRODUCTION			
Rationale	3	Describe the rationale for the review in the context of existing knowledge.	Section 1. Introduction, especially paragraphs 2–5. Discusses the limitations of existing treatments and the potential role of dopaminergic mechanisms in depression.
Objectives	4	Provide an explicit statement of the objective(s) or question(s) the review addresses.	In the last paragraph of the Introduction: "This systematic review aims to evaluate the efficacy and mechanisms of three dopaminergic agents..."
METHODS			
Eligibility criteria	5	Specify the inclusion and exclusion criteria for the review and how studies were grouped for the syntheses.	Section 2.1. Inclusion and Exclusion Criteria
Information sources	6	Specify all databases, registers, websites, organisations, reference lists and other sources searched or consulted to identify studies. Specify the date when each source was last searched or consulted.	Section 2.2. Search Strategy – Lists databases (PubMed, Scopus, Embase, Cochrane, Web of Science, ClinicalTrials.gov, Google Scholar).
Search strategy	7	Present the full search strategies for all databases, registers and websites, including any filters and limits used.	Section 2.2. Search

PRISMA 2020 Checklist

Section and Topic	Item #	Checklist item	Location where item is reported
			Strategy – general keywords mentioned.
Selection process	8	Specify the methods used to decide whether a study met the inclusion criteria of the review, including how many reviewers screened each record and each report retrieved, whether they worked independently, and if applicable, details of automation tools used in the process.	Section 2.3. Eligible Studies – Describes flow from initial search to final inclusion.
Data collection process	9	Specify the methods used to collect data from reports, including how many reviewers collected data from each report, whether they worked independently, any processes for obtaining or confirming data from study investigators, and if applicable, details of automation tools used in the process.	Section 2.4. Data Extraction – States that key study information was extracted.
Data items	10a	List and define all outcomes for which data were sought. Specify whether all results that were compatible with each outcome domain in each study were sought (e.g. for all measures, time points, analyses), and if not, the methods used to decide which results to collect.	Section 2.4. Data Extraction and Tables 1–3 – Implicitly includes depressive symptom measures
	10b	List and define all other variables for which data were sought (e.g. participant and intervention characteristics, funding sources). Describe any assumptions made about any missing or unclear information.	Section 2.4. Data Extraction – mentions study design, drug, dose, duration, route, population.
Study risk of bias assessment	11	Specify the methods used to assess risk of bias in the included studies, including details of the tool(s) used, how many reviewers assessed each study and whether they worked independently, and if applicable, details of automation tools used in the process.	Section 2.5. Data Quality – QATQS tool described; two independent reviewers used.
Effect measures	12	Specify for each outcome the effect measure(s) (e.g. risk ratio, mean difference) used in the synthesis or presentation of results.	Implied in Results (Section 3) via HDRS, MADRS, BDI scores.
Synthesis methods	13a	Describe the processes used to decide which studies were eligible for each synthesis (e.g. tabulating the study intervention characteristics and comparing against the planned groups for each synthesis (item #5)).	Results are presented by drug class – Section 3.1, 3.2, 3.3 (Bromocriptine, Amantadine, Selegiline).
	13b	Describe any methods required to prepare the data for presentation or synthesis, such as handling of missing summary statistics, or data conversions.	All extracted data were reviewed for completeness and consistency. Where summary statistics were not directly reported, relevant values such as response rates or

Section and Topic	Item #	Checklist item	Location where item is reported
			symptom score changes were extracted from narrative descriptions or figures when possible. No imputation or statistical conversion was applied, due to the qualitative nature of the synthesis.
	13c	Describe any methods used to tabulate or visually display results of individual studies and syntheses.	Described in Section 2.4, with presentation in Tables 1–3.
	13d	Describe any methods used to synthesize results and provide a rationale for the choice(s). If meta-analysis was performed, describe the model(s), method(s) to identify the presence and extent of statistical heterogeneity, and software package(s) used.	Synthesis is narrative, no meta-analysis performed.
	13e	Describe any methods used to explore possible causes of heterogeneity among study results (e.g. subgroup analysis, meta-regression).	Given the narrative nature of the synthesis and heterogeneity in study designs, populations, and interventions, subgroup comparisons were conducted qualitatively. Differences in drug formulation (oral vs transdermal), patient characteristics (e.g., treatment-resistant vs atypical depression), and study design (RCT vs observational) were considered as potential sources of heterogeneity and are discussed narratively.
	13f	Describe any sensitivity analyses conducted to assess robustness of the synthesized results.	Formal sensitivity analyses were not performed due to the absence of a meta-analytic approach. However, conclusions were drawn with greater emphasis on findings from randomized controlled

PRISMA 2020 Checklist

Section and Topic	Item #	Checklist item	Location where item is reported
			trials and studies rated as 'strong' in methodological quality, to assess the robustness of observed patterns.
Reporting bias assessment	14	Describe any methods used to assess risk of bias due to missing results in a synthesis (arising from reporting biases).	To minimize the risk of reporting bias, we conducted a comprehensive search across multiple databases and trial registries, including ClinicalTrials.gov. In addition, unpublished studies and case reports were included when available. Due to the narrative nature of the synthesis and the limited number of comparable quantitative outcomes, no statistical assessment of publication bias (e.g., funnel plots) was performed.
Certainty assessment	15	Describe any methods used to assess certainty (or confidence) in the body of evidence for an outcome.	The overall certainty of evidence was assessed qualitatively based on study design, sample size, and methodological quality (as rated using the QATQS tool). Randomized controlled trials were considered more robust than open-label studies or case reports. Although the GRADE approach was not formally applied, consistency of findings and directness of evidence were taken into account when

PRISMA 2020 Checklist

Section and Topic	Item #	Checklist item	Location where item is reported
			interpreting results.
RESULTS			
Study selection	16a	Describe the results of the search and selection process, from the number of records identified in the search to the number of studies included in the review, ideally using a flow diagram.	Section 2.3. Eligible Studies<br>Describes: 1,111 records identified → 600 duplicates removed → 511 screened → 54 full-text reviewed → 28 included.
	16b	Cite studies that might appear to meet the inclusion criteria, but which were excluded, and explain why they were excluded.	Section 2.3. Eligible Studies<br>Mentions 26 studies excluded for lack of relevant outcomes or insufficient relevance.<br>Suggested improvement: Include a table in supplementary materials listing excluded full-text articles with reasons.
Study characteristics	17	Cite each included study and present its characteristics.	Tables 1–3 and detailed in Section 3 (Results)<br>Presents: author, study design, drug used, population, intervention details, and QATQS rating.
Risk of bias in studies	18	Present assessments of risk of bias for each included study.	tables 1–3 – includes QATQS ratings (1–3) per study.<br>Explained in Section 2.5. Data Quality.
Results of individual studies	19	For all outcomes, present, for each study: (a) summary statistics for each group (where appropriate) and (b) an effect estimate and its precision (e.g. confidence/credible interval), ideally using structured tables or plots.	Section 3 (Results) – for each drug, multiple studies are described with changes in scores like HDRS, BDI.<br>Tables 1–3 summarize individual study interventions and responses.

Section and Topic	Item #	Checklist item	Location where item is reported
Results of syntheses	20a	For each synthesis, briefly summarise the characteristics and risk of bias among contributing studies.	In each subsection of Section 3 (e.g., 3.1–3.3). The narrative includes discussion of methodological strength/weakness based on QATQS.
	20b	Present results of all statistical syntheses conducted. If meta-analysis was done, present for each the summary estimate and its precision (e.g. confidence/credible interval) and measures of statistical heterogeneity. If comparing groups, describe the direction of the effect.	no meta-analysis performed. A qualitative synthesis is presented in the text with outcomes from individual studies compared.
	20c	Present results of all investigations of possible causes of heterogeneity among study results.	Addressed indirectly in Discussion section (Section 4) – mentions differences in study populations (age, symptom severity, prior treatment).
	20d	Present results of all sensitivity analyses conducted to assess the robustness of the synthesized results.	No formal sensitivity analyses were performed due to the heterogeneity and narrative nature of the synthesis. However, emphasis was placed on findings from randomized controlled trials when drawing conclusions.
Reporting biases	21	Present assessments of risk of bias due to missing results (arising from reporting biases) for each synthesis assessed.	The risk of reporting bias was considered by including both published and registry-based studies (e.g., ClinicalTrials.gov), as well as case reports and open-label trials. Funnel plot analysis was not applicable due to lack of quantitative

PRISMA

PRISMA 2020 Checklist

Section and Topic	Item #	Checklist item	Location where item is reported
			synthesis.
Certainty of evidence	22	Present assessments of certainty (or confidence) in the body of evidence for each outcome assessed.	No formal GRADE evaluation was performed. Confidence in the evidence was based on study design, sample size, and QATQS ratings. Randomized controlled trials were considered higher certainty compared to open-label and case reports.
DISCUSSION			
Discussion	23a	Provide a general interpretation of the results in the context of other evidence.	Section 4. Discussion – compares findings to earlier research and supports dopaminergic involvement in depression
	23b	Discuss any limitations of the evidence included in the review.	Section 4. Discussion, paragraph 3 – addresses heterogeneity, sample size, short duration, adolescent data limitations.
	23c	Discuss any limitations of the review processes used.	The review process was limited by lack of protocol deviations tracking and the absence of duplicate independent screening and data extraction, which may introduce bias.
	23d	Discuss implications of the results for practice, policy, and future research.	Final paragraph of Section 4 – discusses need for further RCTs, implications for personalized pharmacotherapy, and potential for expanding treatment options.
OTHER INFORMATION			

PRISMA 2020 Checklist

Section and Topic	Item #	Checklist item	Location where item is reported
Registration and protocol	24a	Provide registration information for the review, including register name and registration number, or state that the review was not registered.	Section 2. Materials and Methods – clearly states: “The review protocol was registered in the PROSPERO registry (ID: CRD420251107504).”
	24b	Indicate where the review protocol can be accessed, or state that a protocol was not prepared.	The full protocol is available in the PROSPERO database under the registration number CRD420251107504.
	24c	Describe and explain any amendments to information provided at registration or in the protocol.	No amendments to the original protocol were made during the review process.
Support	25	Describe sources of financial or non-financial support for the review, and the role of the funders or sponsors in the review.	This research received no external funding. The authors declare no financial or institutional sponsorship influencing the review process.
Competing interests	26	Declare any competing interests of review authors.	The authors declare no conflicts of interest.
Availability of data, code and other materials	27	Report which of the following are publicly available and where they can be found: template data collection forms; data extracted from included studies; data used for all analyses; analytic code; any other materials used in the review.	Data extracted from included studies, along with tables used in the synthesis, are available upon reasonable request from the corresponding author. No analytic code was used, as no statistical synthesis was performed.

From: Page MJ, McKenzie JE, Bossuyt PM, Boutron I, Hoffmann TC, Mulrow CD, et al. The PRISMA 2020 statement: an updated guideline for reporting systematic reviews. BMJ 2021;372:n71. doi: 10.1136/bmj.n71. This work is licensed under CC BY 4.0. To view a copy of this license, visit https://creativecommons.org/licenses/by/4.0/
